# Clinical efficacy analysis of cosmetic suture technique combined with tension reducer in the treatment of facial skin trauma

**DOI:** 10.1097/MD.0000000000041040

**Published:** 2024-12-27

**Authors:** Ya Gao, Yalin Wang, Wenbo Li, Fenglian Wu

**Affiliations:** a Department of Surgery, Plastic and Cosmetic Surgery, First Hospital of Qinhuangdao, Haigang District, Qinhuangdao City, Hebei Province, People’s Republic of China; b Hebei North University, Qiaodong District, Zhangjiakou City, Hebei Province, People’s Republic of China.

**Keywords:** cosmetic suture, facial skin trauma, scar hyperplasia, tension reducer

## Abstract

**Background:**

This study aims to observe the clinical efficacy of cosmetic suture technique combined with tension reducer in the treatment of facial skin trauma and provide more sequential treatments for facial skin trauma.

**Methods:**

Sixty patients with facial skin trauma who visited our department from January 2023 to January 2024 were selected as the research subjects. Patients who received cosmetic sutures combined with tension reducers were selected as the observed group (n = 30), while patients who received simple cosmetic sutures were selected as the control group (n = 30). Follow-up at 1, 3, and 6 months after surgery to compare the condition of scar formation (using the Vancouver scar rating scale), scar width, and patient satisfaction between the 2 groups.

**Results:**

After 1, 3, and 6 months of follow-up, the total score of Vancouver scar rating scale in the observed group was lower than that in the control group (*P* < .05); The average postoperative scar width in the observed group was (0.72 ± 0.07 mm), which was narrower than that in the control group (1.03 ± 0.12 mm) (*P* < .05). The satisfaction rate of patients in the observed group was 93.33%, which was higher than 73.33% in the control group (*P* < .05).

**Conclusion:**

The combination of cosmetic sutures and tension reducer in the treatment of facial skin trauma can effectively improve the scar condition, narrow the scar width, and greatly improve patient satisfaction. It is worth popularizing in the treatment of facial skin trauma.

## 1. Introduction

**Figure 1.**
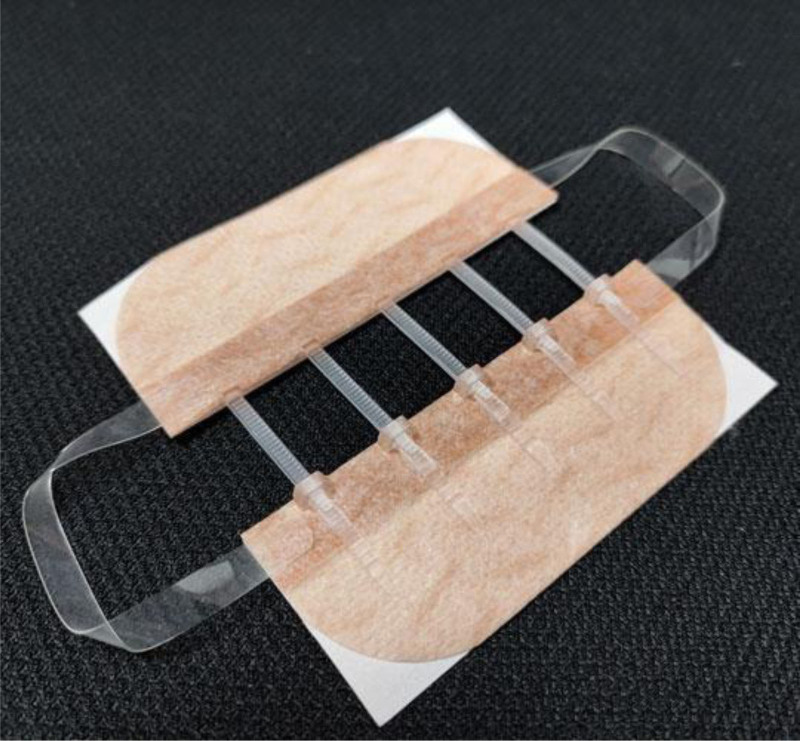
Tension reducer devices.

In people’s daily life, study, and work, it is hard to avoid being injured, and due to the lack of protection such as clothing, the face is prone to varying degrees of facial skin trauma after collisions.^[1]^ With the gradual improvement of people’s living standards and happiness index, the attention to facial appearance is also increasing. Therefore, plastic surgery has received increasing attention in the medical field. In recent years, various general hospitals have gradually launched emergency cosmetic suturing services for plastic surgery, specializing in receiving patients with high prognosis requirements for skin injuries, which are more common in patients with maxillofacial lacerations. The biggest difference between cosmetic sutures and conventional sutures is that the formation of scars is smaller in the prognosis, and even with various sequential treatment methods in the later stage, such as applying anti-scarring drugs,^[2]^ carbon dioxide (CO_2_) laser,^[3,4]^ fluid silicone gels,^[5]^ tension reducing tape,^[6,7]^ and tension reducer, the scar will fade further. Scars are even not obvious to the naked eye. Therefore, how to improve scar prognosis through cosmetic suture technique and choose which later sequential treatment methods has become a hot topic in the field of plastic surgery. This article focuses on the study of using cosmetic suture technique combined with tension reducer to prevention and cure scars in emergency facial skin trauma patients. With the continuous advancement of medical technology, tension reducer devices (Fig. 1) have a better effect on reducing scar after cosmetic suture.^[8]^ Therefore, this study aims to explore the role of skin tension reducer in cosmetic suture.

## 2. Patients and methods

### 2.1. Clinical data

A retrospective analysis was conducted on 60 patients with emergency facial skin trauma who were treated in Qinhuangdao First Hospital Plastic and Cosmetic Surgery from January 2023 to January 2024. The patients were divided into a control group (n = 30) and an observed group (n = 30) based on whether tension reducers were used after cosmetic sutures. The control group consisted of 14 females and 16 males, aged 22 to 40 years, with an average age of 31.2 ± 5.1 years, 9 in the upper face part, 12 in the middle face, and 9 in the lower face part. The wounds were all deep to the fat layer and some deep to the periosteum layer, with an average length of 4.0 ± 0.9 cm. Observed group: among them, there were 13 females and 17 males, aged 18 to 37 years old, with an average age of 29.5 ± 5.7 years old. There were 8 cases in the upper face part, 13 cases in the middle face, and 9 cases in the lower face part. The depth of the wounds reached the fat layer, and some wounds reached the periosteum layer, with an average length of 3.7 ± 0.8 cm. Emergency facial trauma mainly come from walking, falling, and hitting table corners, steps, or windowsills, sports injuries, sharp instrument injuries, and car accidents, causing skin lacerations in different parts of the face. The difference in patient demographics between the 2 groups was not statistically significant (*P* > .05), indicating comparability. The study was approved by the ethics committee of the First Hospital of Qinhuangdao and adhered to the guidelines of the Declaration of Helsinki. Informed consent was obtained from the patient for publication of this case report detail, and patients and their families were informed and signed informed consent.

### 2.2. Inclusion criteria

① Asians aged over 18; ② all wounds are simple laceration and located on the face and have a full layer of skin laceration, with a closure length of at least 3.0 cm. All wounds have no tissue defects or fractures, and there are no important blood vessels, ligaments, facial nerve, or gland damaged inside the wounds; ③ debridement and suturing are performed within 6 to 8 hours after the trauma, and there is no infection and excellent wound healing after surgery; ④ the patient with acute facial skin trauma has stable vital signs; ⑤ regular treatment, regular follow-up, and follow-up was at least 6 months.

### 2.3. Exclusion criteria

① Patients under 18 years old or unable to fully cooperate; ② patients with severe trauma such as combining head, chest, abdominal organ injuries, and fractures; ③ belonging to patients with scar diathesis; ④ photosensitive patients.

### 2.4. Method

#### 2.4.1. Control group

Sign an informed consent before surgery. Disinfect the operative area with iodine, local infiltration anesthesia was performed along the wound margin. Repeatedly irrigate the wound with iodine, saline, and hydrogen peroxide, remove foreign objects inside the wound, correct the skin margin and subcutaneous tissue. Intermittently suture the subcutaneous tissue with 5-0 bioabsorbable thread (Coated VicrylPlus antibacterial suture) and suture the skin epidermis with 7-0 Prolene thread (Ethicon). Regular outpatient follow-up visits will be asked after surgery, wound dressing will be changed the next day, and external thread will be removed 5 to 7 days after surgery. After removing the thread, apply KELO-COTE silica gel externally to the wound twice daily until the last follow-up visit (6 months post-surgery).

#### 2.4.2. Observed group

Using the same suturing method mentioned above. After removing the thread, apply KELO-COTE silica gel externally to the wound twice daily until the last follow-up visit (6 months post-surgery). Immediately use the tension reducer after external thread removal. The specific operation method is as follows: open the aseptic packaging bag of the skin tension reducer, peel off the skin tension reducer from the release paper bottom plate, place both sides of the skin tension reducer parallel to each end of the wound, press the tension reducer to fit tightly into the skin, tighten the 2 sides latchs in sequence to cause slight skin hump at the postoperative wound. Instruct the patient to check the skin tension at the raised area every day to avoid situations where the tension reducer is too loose or excessively tightened, which may affect the effectiveness of the tension reducer. Application time of tension reducer: from the time of external thread removal to 6 months after surgery.

### 2.5. Observation indicators

#### 2.5.1. VSS score

At months 1, 3, and 6 after surgery, scars were scored using the Vancouver scar rating scale (VSS),^[9]^ with a total score of 15 points. The higher the total score, the more severe the postoperative scars.

#### 2.5.2. Scar width

Six months after surgery, measure the scar width at the wound site of 2 groups of patients.

#### 2.5.3. Patient satisfaction

Six months after surgery, the satisfaction of patients after treatment was rated by completing a questionnaire. Satisfaction = (very satisfied + satisfied + acceptable) number of cases/total number of cases × 100%.

#### 2.5.4. Adverse reactions

Observing the occurrence of adverse reactions during the experimental process.

### 2.6. Statistical analysis

SPSS 26.0 statistical program is used to analyze the data. Count data is expressed as percentages (%), chi-square ($x^{2}$) test is used; measurement data is expressed as mean ± standard deviation (±s), *t* test is used for inter group comparison, and the difference is statistically significant with *P* < .05.

## 3. Results

### 3.1. Clinical efficacy comparison

**Figure 2.**
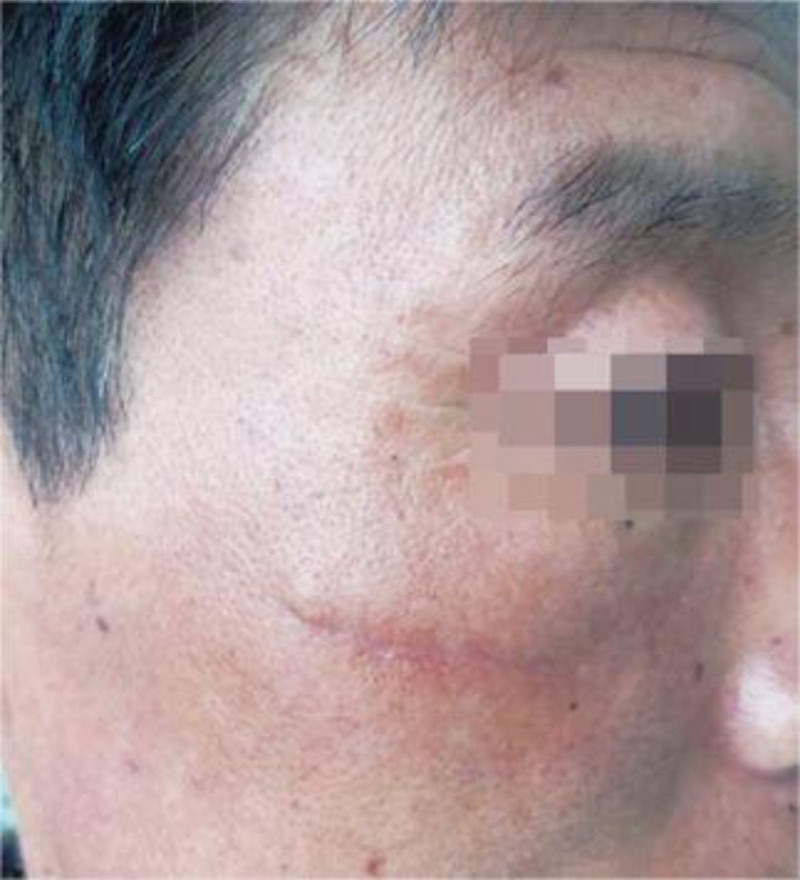
Control group.

**Figure 3.**
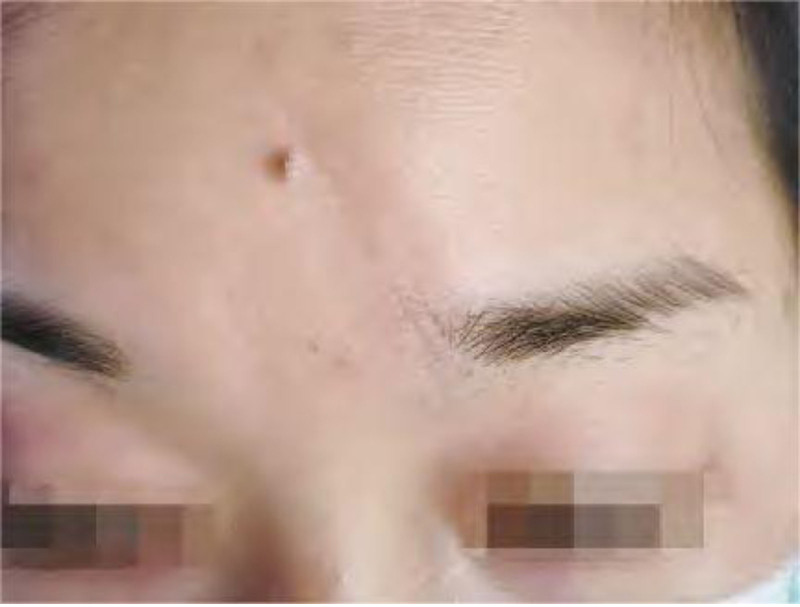
Observed group.

Sixty cases of facial skin trauma all had primary grade A healing, and no hematomas, wound dehiscence, or infection occurred after surgery. After 6 months of surgery, the scars in the observed group were more subtle or even less obvious, while the control group showed light red linear scars, as shown in Figures 2 and 3.

### 3.2. VSS

Comparison of VSS scores at 1, 3, and 6 months after surgery showed that the total VSS score in the observed group was lower than that in the control group, with statistical significance (*P* < .05), as shown in Table 1.

**Table 1:** Comparison of the VSS scores between the 2 groups ($\bar{x}\pm s$, scores).

Groups	Number of cases	1 month after surgery	3 months after surgery	6 months after surgery
Observed group	30	4.23 ± 0.50	2.26 ± 0.58	1.37 ± 0.61
Control group	30	5.13 ± 0.34	3.83 ± 0.59	3.17 ± 0.46
T values		8.065	10.324	12.827
*P* values		<.001	<.001	<.001

### 3.3. Comparison of scar width between 2 groups of wounds

Six months after surgery, the average scar width in the observed group was (0.72 ± 0.07 mm) narrower than that in the control group (1.03 ± 0.12 mm), and the difference was statistically significant (*P* < .05).

### 3.4. Comparison of patients’ satisfaction rates with postoperative scar formation

Six months after surgery, the satisfaction rate of patients in the observed group with the suture effect of facial skin trauma was 93.33%, which was higher than the control group’s 73.33%, and the difference was statistically significant (*P* < .05) (see Table 2).

**Table 2:** Comparison of patient satisfaction between the 2 groups [case (%)].

Groups	Number of cases	Very satisfied	Satisfied	Acceptable	Dissatisfied	Degree of satisfaction (%)
Observed group	30	24	4	1	1	93.33
Control group	30	8	14	2	6	73.33
Chi-square ($x^{2}$) values		17.143	7.937	0.351	4.043	4.320
*P* values		<.001	.005	.554	.044	.038

### 3.5. Adverse reactions

During the experiment, a case of patient developed contact dermatitis due to the use of the tension reducer. The skin at the site of the tension reducer appeared needle sized pale red papules with slight edema, which w ere consistent in range size with the site of the tension reducer. The patient felt itching and burning sensation on the skin. After discovery, go to the author’s department for treatment timely. Remove the tension reducer, rinse the skin thoroughly with normal saline, and apply calamine lotion to the skin after drying. Advise the patient to avoid scratching, sun exposure or hot air stimulation, not to use hot water for flushing, and prohibit the use of the tension reducer. Three days later, the patient was reexamination at the outpatient clinic. The pale red papules on the skin disappeared, the edema faded away compared to before, and the patient reported no itching or burning sensation. This case was not included in the observed group.

### 3.6. Typical case

**Figure 4.**
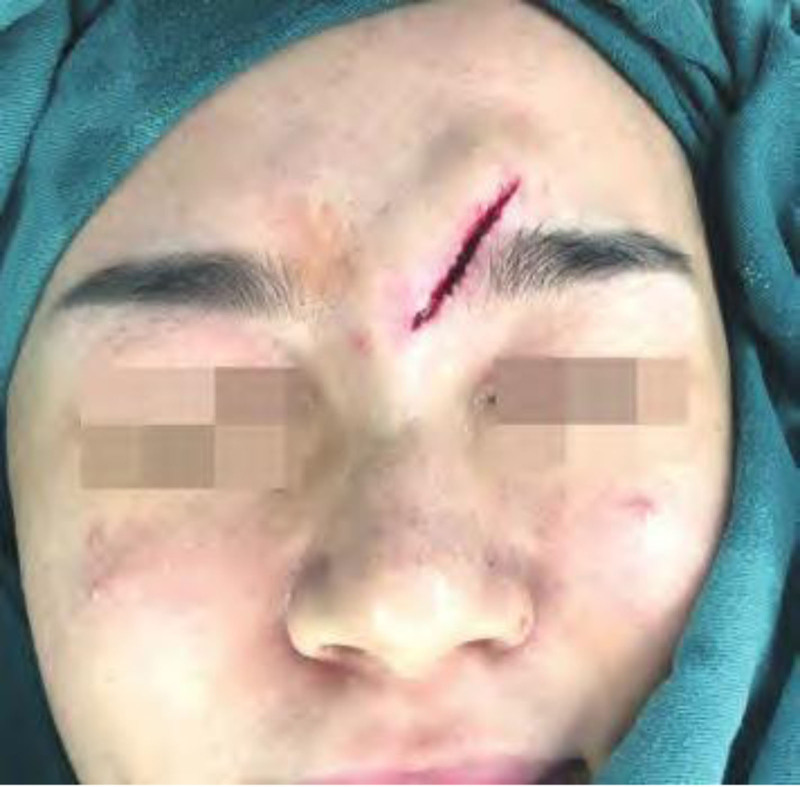
Before surgery.

**Figure 5.**
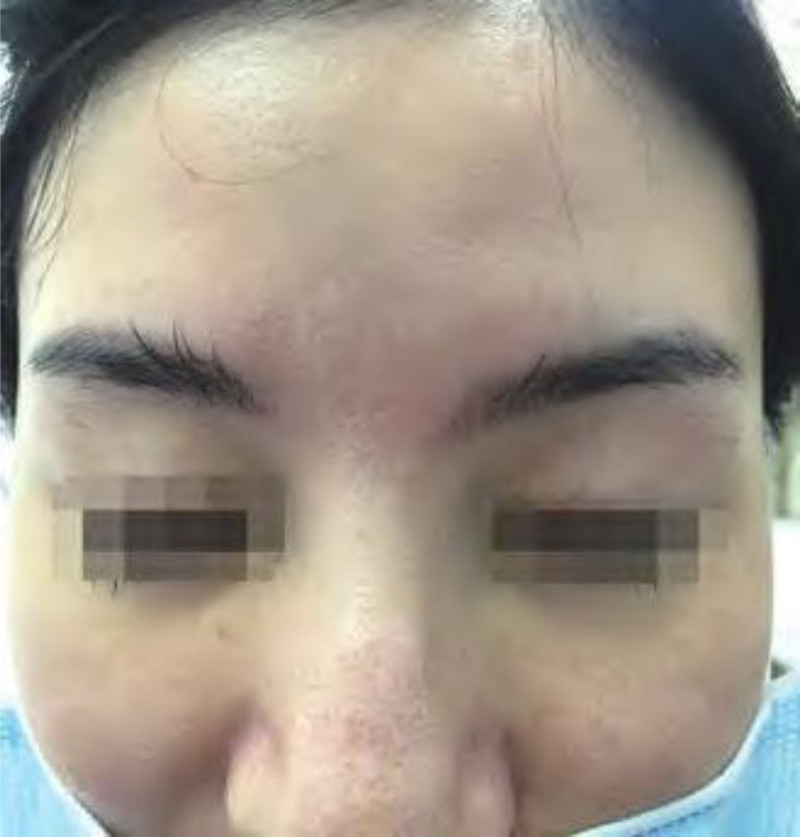
Six months after surgery.

A 26-year-old female presented with an upper face part skin trauma for 1 hour, with a wound measuring 3.2 cm in length and reaching deep into the fat layer (Fig. 4). The emergency surgery was performed using cosmetic suture. Immediately use the tension reducer after external thread removal for 4 months. Follow-up was for 6 months (see Fig. 5).

## 4. Discussion

**Figure 6.**
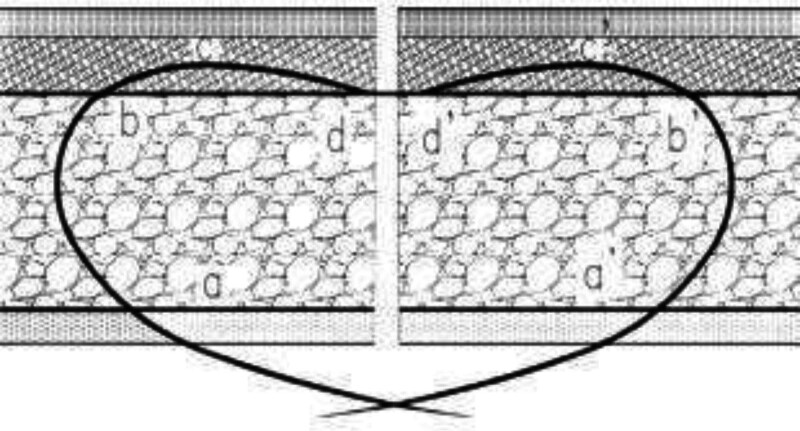
Heart-shaped attension suture.

Traditional ordinary sutures are mostly thick needle and large thread full layer sutures, emphasizing wound alignment, restoring physiological function, and ensuring healing. It is precisely because the thick needle and thread cause significant irritation to the tissue, resulting in a series of problems such as uneven wound edges and high tension in the later stage, ultimately forming wide centipede shaped scars.^[10]^ Therefore, we have abandoned traditional ordinary suturing techniques and opted for more refined cosmetic suturing techniques. The 60 research subjects treated by the author were all treated with subcutaneous heart-shaped attension suture (Fig. 6) ^[11]^ using 5-0 absorbable suture thread to suture the fascia layer, subcutaneous tissue, and dermis layer by layer, forming a minimum tension state in the dermis layer, restoring the position of subcutaneous tissue to the maximum extent, and concentrating the wound tension mostly on the subcutaneous layer. After subcutaneous suture, in order to ensure good alignment of the wound edges on both sides, 7-0 Prolene thread is interruptedly or continuously sutured epidermis layer, so as to achieve the requirements of no tension, no wound residue, no dead space, and prevention of hematoma as much as possible.^[12,13]^ Therefore, in cosmetic sutures, the following points should be noted: (1) polluted wounds should be carefully and thoroughly debridement to avoid infection that can prolong course of disease; (2) the layers of tissue should be accurately aligned, relaxation suture layer by layer, without leaving any dead space. The tension of the wound should be dispersed as much as possible to the subcutaneous tissue and deep dermis, minimizing scars;^[14]^ (3) reasonable selection of suture lines is beneficial for maintaining long-term tension reduction effects.^[15,16]^

Studies have shown that scar hyperplasia last from 3 to 6 months.^[17]^ During the scar hyperplasia period, the scar tissue becomes hard, wide, and congested. Although the absorbable line can be fully absorbed by the physiological tissue within 56 to 70 days, due to individual differences and the inevitable influence of facial expressions and chewing, long-term and repeated muscle movements can easily lead to loose and detached suture lines, causing local tissue displacement and insufficient long-term tension reduction ability of the wound, thereby accelerating the growth of fibroblasts and new vessels. Ultimately, the prognosis of scars cannot meet the patients’ expected results.^[18]^ The design of the latch on both sides of the tension reducer generates a huge lateral traction force, forming a tighter closure of the soft tissues on both sides of the wound, while reducing the surface force of the skin on both sides, significantly reducing the tension on both sides of the wound, facilitating tissue reconstruction, and preventing scar hyperplasia and widening.^[19,20]^ In addition, the detachable latch design of the tension reducer can adjust the tension of the skin incision according to the individual situation of the patient. The strength of the latch can be continuously adjusted as the degree of scar hyperplasia changes.^[8]^ This not only effectively suppresses scar hyperplasia, but also improves the subjective comfort of the patient. It has now become an emerging backbone in the fight against hypertrophic scars.

Among the 60 patients selected by the author, 30 cases in the observed group were treated with a combination of cosmetic sutures and tension reducers to combat long-term scars. The degree of scar hyperplasia at 1, 3, and 6 months after surgery, scar width at 6 months, and patient satisfaction were tracked. The results showed that compared to the group without using tension reducers, the facial scars in the group treated the combination of tension reducers were relatively less obvious, narrower in width, and the patients’ satisfaction was also higher. However, due to the use of tension reducer for at least 3 to 6 months, the treatment process is long, and some patients have poor compliance. In addition, some patients may experience allergic reactions such as skin erythema and papules after using the tension reducer, so further research is needed to benefit more scar patients.

## 5. Conclusion

In summary, the combined use of skin tension reducer after facial cosmetic suture surgery can further improve the healing effect of trauma and reduce scar width, which is worthy of further promotion and application in clinical work.

## Author contributions

**Conceptualization:** Ya Gao, Yalin Wang, Fenglian Wu.

**Data curation:** Wenbo Li.

**Formal analysis:** Ya Gao, Yalin Wang.

**Investigation:** Ya Gao, Yalin Wang, Wenbo Li.

**Methodology:** Ya Gao, Yalin Wang.

**Project administration:** Yalin Wang, Fenglian Wu.

**Supervision:** Fenglian Wu.

**Validation:** Ya Gao, Wenbo Li, Fenglian Wu.

**Writing – original draft:** Ya Gao, Yalin Wang.

**Writing – review & editing:** Fenglian Wu.
